# Deformation Behaviors and Toughening Mechanisms of Gradient-Structured Mg-Gd-Y Alloy

**DOI:** 10.3390/ma18163818

**Published:** 2025-08-14

**Authors:** Bosong Gao, Minghui Wu, Jiangli Ning, Siwei Wang, Yang Wang

**Affiliations:** 1 Innovation Center for New Materials and Processing Technology, Ningbo Institute of Dalian University of Technology, Ningbo 315000, China; 2 North China Aluminium New Material Technology Co., Ltd., Baoding 072750, China

**Keywords:** Mg-RE alloy, gradient structure, in-situ tensile test, deformation mechanism, toughening mechanism

## Abstract

A Mg-Gd-Y alloy prepared by surface mechanical attrition treatment (SMAT) was annealed at 450 °C combined with peak aging. The deformation and fracture mechanisms were investigated using in situ tensile tests. Through quantitative calculations of the geometrically necessary dislocation (GND) densities, it was found that the fine-grained (FG) layer in the gradient structure carried greater plastic strain than the coarse-grained (CG) layer during tension. The calculation results of the geometric compatibility parameter (m’) and microstructure characterization during in situ tests showed that crack initiation and propagation were prone to occur between adjacent coarse grains. However, the hetero-deformation-induced (HDI) strengthening and strain hardening induced by the strain gradient between the FG and CG layers effectively improved the strength–ductility synergy of the gradient-structured (GS) alloy. In addition, the synergistic effect of intrinsic and extrinsic toughening mechanisms in the GS alloy played a significant role in delaying premature failure.

## 1. Introduction

Although magnesium has the advantages of a low density and high specific strength, its shortcomings of inadequate absolute strength, low plasticity, and poor sustainability are still obstacles for practical applications. Alloying is generally regarded as an effective method to improve the mechanical properties of magnesium alloys. Previous studies [1,2,3] have shown that adding Gd and Y elements to the Mg matrix can improve yield strength and plasticity, as well as weaken the internal texture of the material. The addition of these rare earth elements can cause solid-solution effects and form strengthening phases [4,5,6]. Thereby, Mg-RE alloys have attracted widespread attention as potential lightweight industrial materials.

In order to obtain high-performance Mg-RE alloys, various deformation processes and heat treatments have been used to enhance their mechanical properties. During the high-temperature deformation of Mg–RE alloys, dislocation slide serves as the dominant rate-controlling mechanism, while mechanical twinning and dynamic recrystallization significantly influence the flow behavior and microstructural evolution of alloys [7]. In recent years, it has been found that introducing heterostructures can effectively improve the strength and ductility of metallic materials [8,9,10,11]. Gradient-structured (GS) materials have attracted significant research interest due to their special strengthening mechanisms and the potential to achieve strength–ductility balance. At present, gradient structures are generally prepared by surface mechanical grinding treatment (SMGT) [12], surface mechanical attrition treatment (SMAT) [13], shot peening [14], torsion deformation [15], friction stir processing (FSP) [16], etc. Among them, SMAT has significant advantages due to the high efficiency, low cost, and industrial adaptability of preparing GS materials.

However, although the strength of SMATed magnesium alloys is improved due to surface nanocrystallization, their ductility is generally restricted [17,18]. Moreover, after aging treatment, the coarse grains in magnesium alloys can exhibit brittle fracture modes, leading to a low global ductility of GS magnesium alloys containing precipitated phases [19,20,21,22]. Previous studies have shown [21,23,24] that recrystallized fine grains can mitigate the brittle cracking of magnesium alloys induced by precipitates, thus improving the strength–ductility synergy of the material. Furthermore, in grain hetero-structure materials, geometrically necessary dislocations (GNDs) accumulate at the coarse- and fine-grain interfaces during plastic deformation, resulting in hetero-deformation-induced (HDI) strengthening [25]. Therefore, constructing a gradient structure combining recrystallized fine grains and original coarse grains could provide an avenue to avoid the catastrophic failure caused by coarse-grained (CG) structures with precipitations [22]. However, the deformation behaviors and toughening mechanisms of this kind of GS magnesium alloy, especially the interactive and individual roles of the constituted layers, have not been thoroughly studied.

This work employed in situ experimental methods to present a detailed study on the deformation and fracture behaviors of a GS Mg-8.75Gd-2.85Y alloy processed by SMAT followed by annealing and peak aging treatment, in order to elucidate the underlying ductilization and toughening mechanisms. It provides insights for the optimization of internal microstructural design in GS Mg-RE alloys. The 8.75Gd-2.85Y ratio was chosen based on established Mg-Gd-Y phase diagrams indicating peak β’ phase formation at 8-9 wt% Gd, with Y additions of >2.5 wt% enhancing corrosion resistance through impurity neutralization [26].

## 2. Materials and Methods

The Mg-8.75Gd-2.85Y (wt%) alloy in this experiment was prepared with raw materials of high-purity magnesium, Mg-25Gd (wt%) and Mg-25Y (wt%) intermediate alloys. The alloy was melted in a low-carbon steel crucible at 750 °C under a protective atmosphere consisting of CO_2_ and SF_6_ mixed at a ratio of 100:1. After casting, an ingot with a diameter of 125 mm and a height of 200 mm was obtained. The actual chemical composition of the ingot was measured by an inductively coupled plasma-atomic emission spectrometer (ICP-AES, GOLD, Chongqing, China), which was Mg-8.75Gd-2.85Y (wt%). The ingot was subjected to homogenization treatment at 500 °C for 12 h, followed by forced-air cooling. Subsequent extrusion was performed at a temperature of 450 °C with an extrusion ratio of 16:1 and an extrusion rate of 0.3 mm/s.

Disk-shaped specimens (Ø49 mm × 1 mm) were subjected to solution heat treatment at 500 °C for 4 h to achieve a homogeneous coarse-grained (CG) microstructure. N_2_ served as a protective atmosphere to prevent oxidation of the samples. The gradient structure was then introduced via SMAT using an SPEX 8000M Mixer/Mill (SPEX SamplePrep, Metuchen, NJ, USA) with stainless steel balls at a frequency of 50 Hz for 60 min. This frequency ensured that the ball milling medium (stainless steel balls) obtained sufficient kinetic energy to impact the surface of the samples. A frequency that was too low would not provide sufficient impact energy to effectively break down coarse grains; an excessive frequency could lead to overheating, sample oxidation, and increased wear and tear of equipment. A processing time of 60 min was crucial for achieving the desired gradient structure depth and a stable state of the fine-grain (FG) layer. High-temperature short-term annealing can effectively eliminate deformation-induced stresses while minimizing grain coarsening, thus, the GS samples were annealed at 450 °C for 1 min and subsequently quenched in water at 70 °C to relieve the high stress concentration and facilitate the formation of fine recrystallized grains in the deformed layer. Peak aging treatment was performed at 225 °C for 20 h, with the samples immersed in silicone oil to ensure uniform heating. The sample subjected to the procedure of extrusion + solid solution + SMAT+ annealing + peak aging was labeled as G450. For comparison, a homogeneous CG sample (HCG) was prepared by a procedure of extrusion + solid solution + peak aging, using identical parameters but without SMAT.

Microstructure characterization was conducted on longitudinal sections of the specimens, which were mechanically polished and etched with a 4% nitric acid-alcohol solution prior to optical microscopy observation. Conventional tensile tests were conducted on an Instron-3382 tester (Instron Corporation, Boston, MA, USA) equipped with a video extensometer (AVE 2^®^, Instron Corporation, Boston, MA, USA) at a loading rate of 5 × 10^−4^ s^−1^. The tensile specimens had gauge dimensions of 10 mm × 3 mm × 0.7 mm for both HCG and G450 samples. The sample dimensions were determined according to ISO 6892:1998–03, Metallic Materials—Tensile Testing at Ambient Temperature. To minimize the surface roughness caused by SMAT, approximately 50 µm of the severely deformed surface layer was removed by mechanical grinding. All specimens were oriented such that their longitudinal axes aligned with the extrusion direction (ED). The tests were repeated at least 3 times for each condition to ensure reproducibility. Vickers hardness tests were also performed on the G450 samples, measured using a Stucker hardness tester under a 25 g load for 8 s. Each reported final hardness value represents the mean of 7 individual test points.

Furthermore, G450 specimens with a gradient structure were used for in situ tensile tests to illustrate the interactions between FG and CG layers. Dog-bone-shaped tensile specimens with a 3 mm gauge length, 2 mm width, and 0.7 mm thickness were prepared with their longitudinal direction parallel to the ED. In situ tensile tests were conducted at room temperature under a constant displacement rate of 1 μm/s. Microstructure evolution during deformation was monitored through intermittent scanning electron microscopy (SEM, ZEISS GeminiSEM 300, Munich, Germany) and electron backscatter diffraction (EBSD, Oxford NordlysMax3, Oxford, UK) analyses at progressively increasing strain levels, with EBSD maps acquired using a step size of 1 μm and processed through Channel 5 (v5.0.9.0). Prior to EBSD testing, the samples were mechanically ground and electropolished in AC2 electrolyte at 20 V for 90 s.

## 3. Results

### 3.1. The Gradient Structure and Hardness Distribution

**Figure 1:**
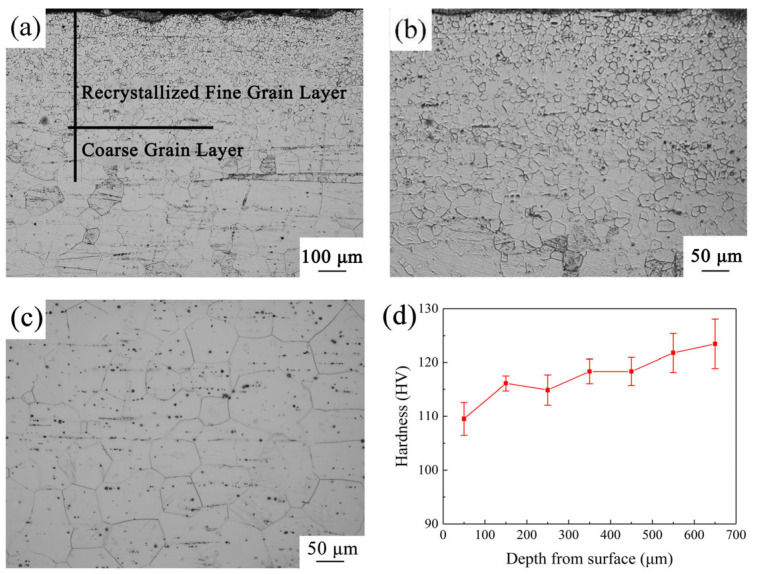
The optical micrographs of (**a**) the G450 sample, (**b**) the magnified view of the recrystallized fine grain layer, and (**c**) the HCG sample; (**d**) the Vickers hardness distribution along the depth direction of the G450 sample.

Figure 1 shows an optical micrograph and the hardness distribution of the longitudinal section of the G450 sample, as well as a micrograph of the HCG sample for comparison. Figure 1a exhibits a gradient grain structure comprising a recrystallized FG layer and a CG layer. Additionally, the recrystallized FG layer was largely devoid of coarse secondary phases at grain boundaries, whereas the unrecrystallized CG layer contained prominent dark blocky secondary-phase particles along grain boundaries (Figure 1a) and finer intragranular precipitates. The magnified view in Figure 1b reveals the morphology of the recrystallized FG layer, further confirming the general absence of coarse boundary phases and a relatively clean microstructure with finer grains. The average grain size of the two different types of grains was calculated by an image processing software (Image J v1.53t). Quantitative analysis presented an average grain size of 10.3 μm for the recrystallized FG region, while the unrecrystallized CG region showed a markedly larger grain size of 76.5 μm.

Figure 1c shows the grain structure of the HCG sample, with a similar grain size (78.6 μm) to the CG region in the G450 sample. The microstructure of the HCG sample shows a highly uniform distribution of small dark precipitates throughout the entire grain. This distribution is in sharp contrast to the obvious phase distribution observed in the G450 sample. Figure 1d presents the hardness distribution along the depth direction in the G450 sample, showing an inverse gradient trend, which is in line with a previously reported GS Mg-Gd-Y alloy processed by a similar procedure. This previous investigation demonstrated that the reduced hardness in the FG layer relative to the CG layer was primarily attributed to diminished particle strengthening. The observed differences in phase distribution (with fewer coarse boundaries in the FG layer and the widespread presence of fine precipitates in the CG layer and HCG sample) provide direct microstructural evidence supporting this explanation. Specifically, the abundance of strengthening precipitates (fine particles) within the grains in the CG region and the HCG sample likely contributed significantly to their higher hardness, while their relative scarcity in the FG layer reduced this strengthening contribution, despite its finer grain size. This difference in strengthening contribution has been confirmed as the main cause of the reverse hardness gradient [22].

### 3.2. Tensile Properties

**Figure 2:**
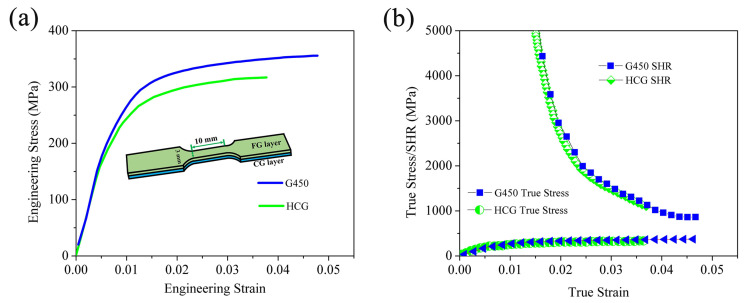
(**a**) Engineering stress–strain curves of the HCG and G450 samples and (**b**) the true stress–true strain and the strain hardening rate curves.

Figure 2a shows the engineering stress–strain curves of the HCG and G450 samples. Their corresponding mechanical properties and some similar Mg-RE alloys reported in the literature [27,28,29] are summarized in Table 1. Compared to the HCG sample and Mg-RE alloys in other works, the G450 sample demonstrates a superior strength–ductility synergy. This enhancement primarily originates from the coordinated deformation behavior between fine- and coarse-grained layers within the gradient architecture, which enables simultaneous strengthening and toughening effects. The detailed microstructural evolution during deformation will be systematically investigated through in situ tensile testing in the following sections. Figure 2b reveals the strain hardening behaviors of the samples. Prior to fracture, the strain hardening rate (SHR) curves of both the HCG and G450 samples exhibited similar trends: an initial sharp decline followed by a gradual decrease. Notably, neither curve intersected the true stress curve, indicating that failure occurred before necking initiation [30]. However, the G450 sample approached closer to the necking point compared to the HCG sample, suggesting enhanced ductility and delayed fracture.

Based on the above, the elongations of the samples were related to the uniform plastic deformation governed by their strain hardening capabilities, as well as their fracture behaviors. In the present GS alloy, these were closely related to the architectural heterogeneous structure, in association with the deformation and fracture mechanisms [31]. This necessitates detailed investigations of (1) the micromechanism of the deformation during tension, particularly the dislocation activities in the gradient structure, and (2) the fracture and toughening mechanisms, in consideration of premature failure significantly impacting overall ductility.

### 3.3. In Situ Observation of the Deformation and Fracture

**Figure 3:**
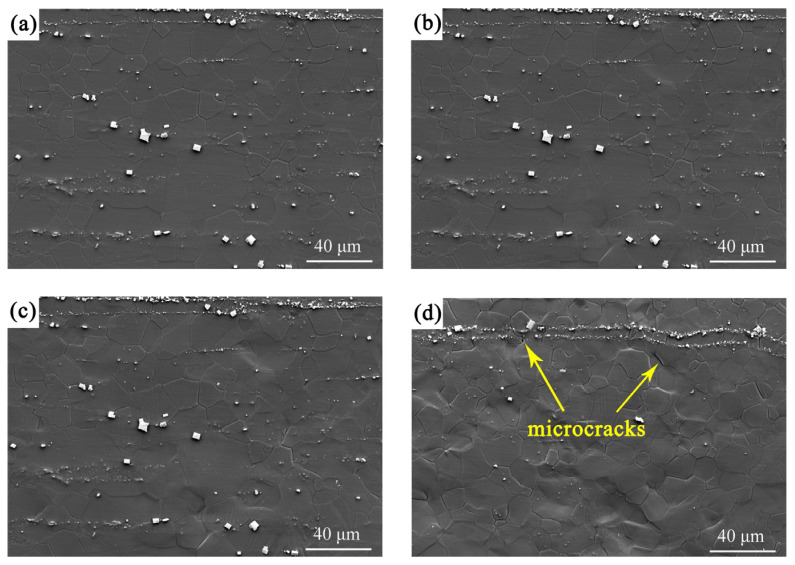
Representative deformation microstructures of the FG region in the G450 specimen at different tensile strains: (**a**) 0.43%, (**b**) 1.29%, (**c**) 2.28%, and (**d**) 3.67%.

Figure 3 presents the microstructure evolution of the recrystallized FG region with progressively increasing tensile strains. At the initial strain of 0.43% (Figure 3a), the microstructure maintained a relatively smooth and uniform morphology. As strain increased to 1.29% (Figure 3b) and further to 2.28% (Figure 3c), the specimen surface showed an increasing roughness, indicating the onset of plastic deformation. At the highest strain of 3.67% (Figure 3d), the surface morphology became markedly uneven, accompanied by the formation of microcracks, demonstrating the progression of damage accumulation prior to fracture.

**Figure 4:**
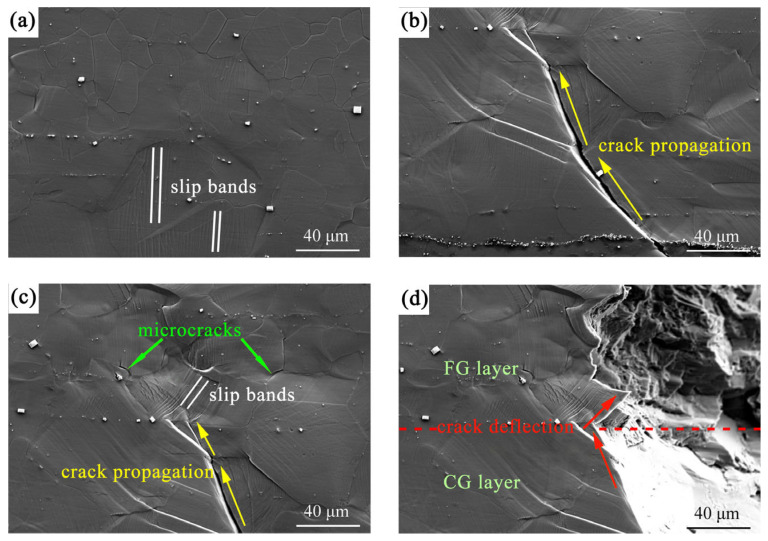
Microstructure of the G450 sample at the interfacial region between the FG and CG layers at different tensile strains: (**a**) 1.29%, (**b**) 3.67%, (**c**) 3.67%, and (**d**) 3.72%.

Figure 4 illustrates the microstructure evolution at the interfacial region between the FG and CG layers in the G450 sample with increasing tensile strains. At a strain of 1.29% (Figure 4a), distinct slip bands became evident within the coarse grains. When the strain reached 3.67% (Figure 4b), a major crack initiated in the CG region and propagated longitudinally through the microstructure. The crack propagation path exhibited significant deflection as it penetrated into the FG region (Figure 4c), primarily due to variations in grain size and misorientation angles between the adjacent grains [19,32]. This could hinder the progression of the major crack. Final fracture occurred at a strain of 3.72% (Figure 4d), showing distinct fracture surface morphologies: the CG region showed a relatively smooth surface indicative of brittle fracture, while the FG region displayed a zigzag pattern characteristic of ductile fracture.

**Figure 5:**
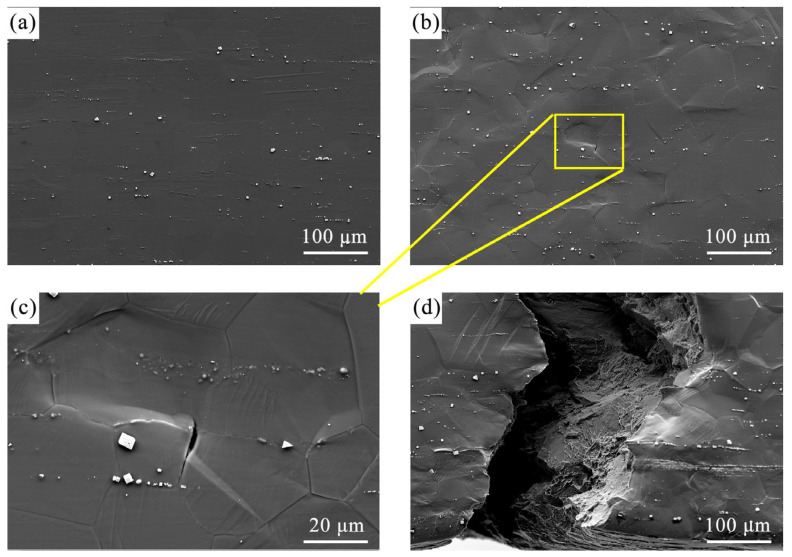
Microstructure of the HCG sample at different tensile strains: (**a**) 0%, (**b**) 2.62%, (**c**) the magnified view of the yellow rectangle in (**b**,**d**) 3.69%.

Figure 5 presents the microstructure evolution of the HCG sample with a progressively increasing tensile strain. The initial microstructure prior to deformation (Figure 5a) exhibited a smooth, homogeneous surface morphology. With an increasing strain, significant surface roughening developed (Figure 5b), indicative of plastic deformation among grains. A magnified view exhibited the microcrack initiation along the grain boundary (Figure 5c). Final fracture occurred at a strain of 3.69%, with the fracture surface exhibiting cracking along grain boundaries or cleavage facets, characteristics of brittle fracture features.

### 3.4. Strain Partitioning in the Gradient Structure

**Figure 6:**
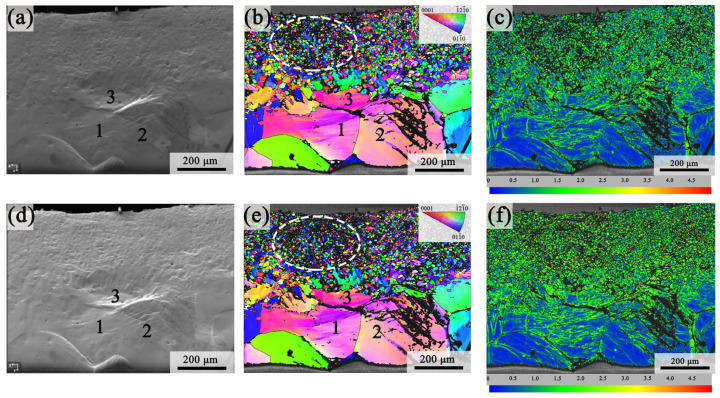
The SEM images, IPF maps, and KAM maps at strains of (**a**–**c**) 2.67% and (**d**–**f**) 3.02%. The numbers 1, 2, and 3 correspond to the different positions in the CG region.

Figure 6 presents the SEM, inverse pole figure (IPF), and kernel average misorientation (KAM) images of the G450 specimen at different tensile strains (2.67% and 3.02%) during in situ tensile testing. As shown in Figure 6a,d, an increasing tensile strain led to the formation of apparent slip traces in the CG region. Notably, the coarse grains 1, 2, and 3 exhibited severe deformation. They provided the potential crack initiation sites, which will be discussed in Section 4.1. From Figure 6b,e, it is evident that the recrystallized FG region experienced increasing distortion due to strain concentration (indicated by the black undefined pixels denoted by white dashed circles). Figure 6c,f display the corresponding KAM maps at tensile strains of 2.67% and 3.02%, respectively. These maps reveal that the plastic strain in the recrystallized FG region was significantly higher than that in the CG region.

**Figure 7:**
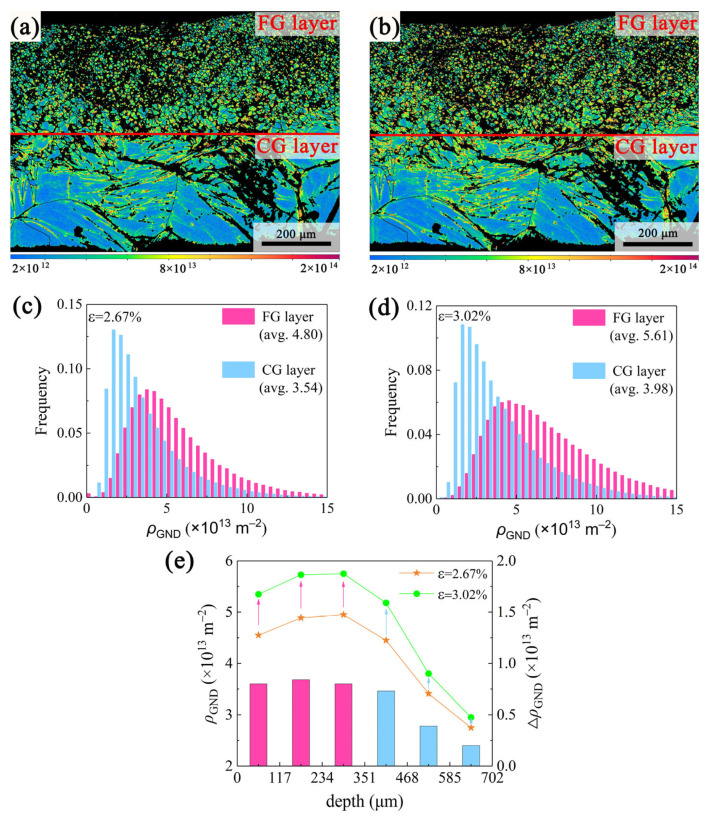
Geometrically necessary dislocation (GND) density maps of the G450 specimen at tensile strains of (**a**) 2.67% and (**b**) 3.02% and the corresponding GND density frequency distributions in the FG and CG layers at strains of (**c**) 2.67% and (**d**) 3.02%; (**e**) the values and increment of GND density at different depths as strain increases from 2.67% to 3.02%.

Figure 7 presents a quantitative analysis of the GND density in the G450 specimen at 2.67% and 3.02% strains. The GND distributions in the FG and CG layers are illustrated in Figure 7a,b. The distributions of the GND density frequency in the FG and CG regions at different strains are presented in Figure 7c,d, respectively. With an increasing strain, the average GND density in the FG region rose from 4.80 × 10^13^ m^−2^ to 5.61 × 10^13^ m^−2^, whereas the CG region showed an increase from 3.54 × 10^13^ m^−2^ to 3.98 × 10^13^ m^−2^. Figure 7e depicts the variation in GND density with depth (dotted lines) at different strains; meanwhile, the pink and blue histograms represent the GND density increments in the FG and CG layers, respectively. Notably, both the GND density and its increments were markedly higher in the FG region than in the CG region, indicating that the FG region played a more significant role in accommodating plastic strain during tensile deformation [33,34]. Furthermore, the synergistic combination of an elevated GND density and grain refinement in the FG region led to more significant dislocation strengthening and grain boundary strengthening, resulting in markedly higher strengthening compared to the CG region.

## 4. Discussion

### 4.1. Strain Coordination and Crack Initiation

The strain coordination between adjacent grains is generally described by a geometric compatibility parameter (*m’*). It represents the geometric match of two adjacent deformation systems, as shown in Equation (1) [35], as follows:

*m*’ = cos*ψ* cos*к* (1)

where *ψ* is the angle between the slip direction of the adjacent grains and *κ* is the angle between the normal slip planes of the adjacent grains.

**Figure 8:**
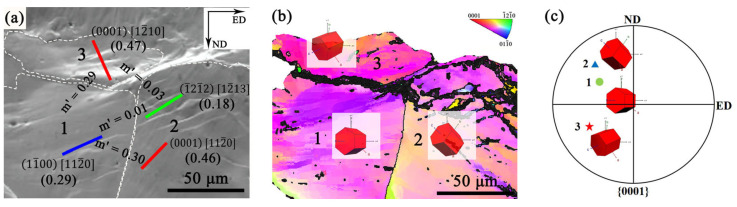
The m’ analysis of regions prone to crack initiation in the G450 specimen: (**a**) SEM image at strain of 3.02%, where the solid red, blue, and green lines represent slip traces; (**b**) the IPF map of grains 1, 2, and 3; and (**c**) the corresponding pole figure at strain of 3.02%.

As shown in Figure 8, the strain compatibilities between grains 1, 2, and 3 in the CG region of the G450 specimen were analyzed at a strain of 3.02%. The Euler angles for grains 1, 2, and 3 were (89.03°, 146.05°, 30.32°), (46.52°, 127.33°, 16.08°), and (114.03°, 130.71°, 13.04°), respectively. According to the slip trace analysis, grain 1 was identified to perform prismatic slip, in which the activated slip system was ($1\bar{1}00$) [$11\bar{2}0$] and the Schmid Factor (SF) was 0.29. Grain 2 was determined to have multiple slips, in which the slip systems were (0001) [$11\bar{2}0$] and ($\bar{1}2\bar{1}2$) [$1\bar{2}13$]. The corresponding SFs were 0.46 and 0.18, respectively. Grain 3 was identified to perform basal slip, in which the activated slip system was (0001) [$1\bar{2}10$] and the SF was 0.47.

It is noted that slip transfer is more likely to occur when the *m’* value is relatively high (>0.7) [36]. As shown in Figure 8a, the *m’* values between the grain pairs (1 and 2), (1 and 3), and (2 and 3) were *m’* = (0.01, 0.30), *m’* = 0.29, and *m’* = 0.03, respectively. All the *m’* values were low, implying that the poor strain compatibility between adjacent CGs could lead to stress concentration and a tendency for crack initiation during tensile deformation [36,37]. This is consistent with the crack formation within the region of CGs, as illustrated in Figure 4b,c.

### 4.2. HDI Strengthening and Strain Hardening

Gradient structures are beneficial for strengthening and strain hardening, arising from the strain gradient generated by the heterogeneous deformation of the soft and hard regions upon loading. Quantitative analysis of GND densities (Figure 7e) revealed that the FG region of the G450 sample exhibited both a higher GND density and larger increment upon straining compared to the CG region. This suggests that the recrystallized FG layer, acting as the soft region, participated in accommodating significant plastic strain, while the CG layer served as the hard region, providing strength. The discrepancy in mechanical responses between the two regions caused strain incompatibility, leading to pronounced strain gradients [38].

The variation in GND densities with depth from the surface (Figure 7e) demonstrated that those in the FG layer increased with depth until reaching the interface with the CG layer, across which they began to decline. This indicates that the strain gradients near the interface between the FG and CG layers were accommodated by the accumulation of GNDs. Tensile test results (Figure 2a) confirmed that the G450 sample possessed a higher yield strength and improved elongation compared to the HCG sample. These observations suggest that the GND accumulations near the interface between the two layers induced back stress in the FG layer (soft region) and forward stress in the CG layer (hard region), effectively activating HDI strengthening and strain hardening. The HDI strengthening contributed to the elevated yield strength, and the HDI strain hardening helped to sustain the uniform deformation [25].

### 4.3. Intrinsic and Extrinsic Toughening Mechanisms

According to recent developments in fracture mechanics, the toughening mechanisms of materials can be categorized into intrinsic and extrinsic toughening [39]. Intrinsic toughening represents the inherent ability of materials to resist crack nucleation and propagation, which is closely associated with microstructural characteristics including grain size, morphology, and second-phase particle distribution. This mechanism enhances the damage resistance of materials by promoting plastic zone expansion at crack tips [40,41]. During tensile deformation, the G450 specimen developed GNDs near cracks to accommodate localized stress concentrations. These GNDs not only relieved stress, but also effectively inhibited crack propagation. As external load increased, the initial plastic zone progressively expanded into a large region, with GNDs playing a crucial role in this process [42]. As the major crack extended with the strain increasing, the plastic zone extended from the stress-concentrated CG region into the FG region. This expansion involved significant energy dissipation through dislocation interactions and movement. Importantly, the generation and accumulation of GNDs enhanced material toughness by slowing the crack propagation rate. The combined effects of plastic zone expansion and GND-mediated deformation contributed substantially to the intrinsic toughening of the material.

**Figure 9:**
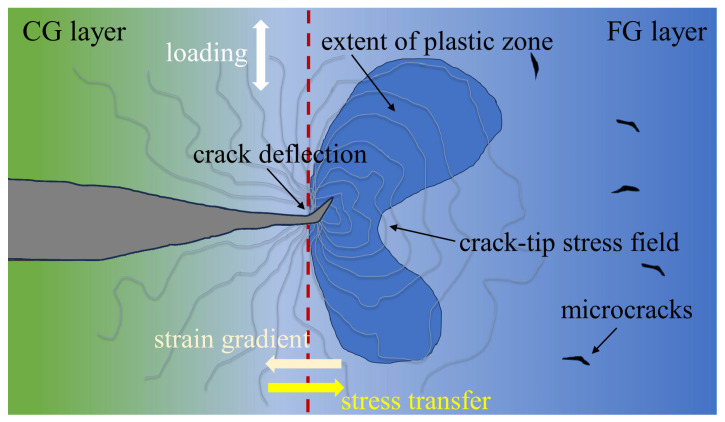
Schematic diagram of intrinsic and extrinsic toughening mechanisms.

A plastic zone of size r*_yi_* developed near the newly formed crack tip, which can be characterized by Equation (2) [43], as follows:

(2) $$r_{yi}=\frac{1}{2\pi }\;{\left(\frac{K_{I}}{\sigma_{yi}}\right)}^{2}$$

where *r_yi_* represents the plastic zone size of the *i*-th layer, *K_I_* is the stress intensity factor, and *σ_yi_* denotes the yield strength of the *i*-th layer. As evidenced in Figure 1d, the G450 sample exhibited a higher Vickers hardness in its CG layer compared to the FG layer. Consequently, the plastic zone in the CG region could be smaller than that in the FG region. Thereby, when the crack propagated from the CG region to the FG region, the expansion of the plastic zone radius effectively mitigated the stress concentration, as demonstrated in Figure 9. Thus, the intrinsic toughening mechanism of the G450 sample substantially enhanced the toughness through strategic plastic zone regulation.

Extrinsic toughening operates by mitigating the local stress near the crack tips through mechanisms such as crack deflection and bridging, thereby reducing the driving force of crack propagation [44,45]. In GS materials, these extrinsic mechanisms become particularly pronounced due to geometric constraint effects introduced by microstructural heterogeneity [46]. This is ascribed to the stress/strain partitioning between the different regions upon loading. The G450 sample created distinct mechanical response zones: the CG region provided a higher yield strength, while the FG region offered better plasticity. Such heterogeneity in properties could readily induce strain gradient and stress transfer between the two layers and consequently lead to stress relaxation, resulting in retarding of the crack propagation [31].

As demonstrated in Section 4.1, the primary crack preferentially initiated and propagated between adjacent coarse grains. When it propagated into the FG region, fine grains with high-density grain boundaries impeded crack advancement, forcing cracks to deflect along grain boundaries (Figure 4d). This deflection generated tortuous crack paths that dissipated additional propagation energy, thus enhancing material toughness [19,31,40]. Moreover, the G450 samples exhibited different fracture modes: brittle fracture in the CG region and ductile fracture in the FG region (Figure 4d). The fracture morphology of the FG region could be ascribed to the activities of non-basal slip systems due to the small grain sizes [19]. This was demonstrated by the slip systems in the fine grains near the crack tip (Figure 4c). The transformation from a brittle to ductile crack also caused deflection of the crack front, slowing down the crack propagation and resulting in toughening.

## 5. Conclusions

In this study, the deformation behavior and toughening mechanisms of a GS Mg-Gd-Y alloy were systematically investigated. The key findings can be summarized as follows:

(1) The developed strain gradient between the FG and CG layers effectively activated the HDI strengthening mechanism and enhanced the strain hardening capability, leading to simultaneous improvements in both the strength and ductility of the GS alloy.

(2) In situ observations combined with geometric compatibility calculations revealed that cracks preferentially initiated and propagated between adjacent coarse grains. Quantitative analysis of dislocation behaviors revealed that the GND density and its strain-induced increment were consistently higher in the FG region than in the CG region, revealing a crucial role of the FG layer in accommodating plastic deformation during the tensile straining of the GS sample.

(3) The gradient structure exhibited significant fracture resistance through the following two complementary mechanisms: intrinsic toughening via adaptive plastic zone expansion that relieved crack tip stress concentrations, and extrinsic toughening through crack deflection and stress/strain partitioning between the hard and soft regions. The synergistic interaction of these two mechanisms significantly postponed crack advancement.

## Figures and Tables

**Table 1:** Tensile properties of the HCG and G450 samples and Mg-RE-based alloys in reference [27,28,29].

	Tensile Strength/MPa	Yield Strength/MPa	Elongation/%
HCG (This work)	321.7 ± 11.4	225.0 ± 6.1	3.7 ± 0.3
G450 (This work)	355.9 ± 15.5	266.2 ± 10.0	4.8 ± 0.6
Mg-Gd-Y-Zr [27]	348	237	3
Mg-Gd-Y-Zr [27]	290	192	13
Mg–Gd–Y–Nd–Zn–Zr [28]	271 ± 5.7	143 ± 3.5	18.7 ± 0.7
Mg-Gd-Y-Zn-Zr [29]	339	299	3.9

## Data Availability

The original contributions presented in this study are included in the article. Further inquiries can be directed to the corresponding authors.
